# Recent Advances of Photocatalytic Application in Water Treatment: A Review

**DOI:** 10.3390/nano11071804

**Published:** 2021-07-12

**Authors:** Guangmin Ren, Hongtao Han, Yixuan Wang, Sitong Liu, Jianyong Zhao, Xiangchao Meng, Zizhen Li

**Affiliations:** Key Laboratory of Marine Chemistry Theory and Technology, Ministry of Education, College of Chemistry and Chemical Engineering, Ocean University of China, Qingdao 266100, China; 17568022691@163.com (G.R.); hanhongtao1998@163.com (H.H.); wyx97921@163.com (Y.W.); liusitong963@163.com (S.L.); jianyongz1222@163.com (J.Z.); mengxiangchao@ouc.edu.cn (X.M.)

**Keywords:** photocatalysis, wastewater treatment, semiconductors, heavy metal, disinfection

## Abstract

Photocatalysis holds great promise as an efficient and sustainable oxidation technology for application in wastewater treatment. Rapid progress developing novel materials has propelled photocatalysis to the forefront of sustainable wastewater treatments. This review presents the latest progress on applications of photocatalytic wastewater treatment. Our focus is on strategies for improving performance. Challenges and outlooks in this promising field are also discussed. We hope this review will help researchers design low-cost and high-efficiency photocatalysts for water treatment.

## 1. Introduction

Over the last few decades, due to population growth and rapid industrialization, ubiquitous contamination includes organic pollutants, heavy metals, inorganic compounds, and many other complex compounds have been detected in surface, ground, sewage and drinking water resources [1]. According to the United Nations’ World Water Development Report (2020) [2], changes in the water cycle will also pose risks to energy production, food security, human health, economic development and poverty reduction, seriously jeopardizing the achievement of sustainable development goals. Therefore, it is essential to develop advanced, environmentally friendly, low-cost and high-efficiency reclamation of wastewater.

There are many different technologies applied in wastewater decontamination, primarily including electrodialysis [3], membrane filtration [4], precipitation [5], adsorption [6], electrochemical reduction [7], and electrodeionization [8]. These processes usually consume large amounts of energy and may be more complicated by transferring pollutants between different fluids, various wastes, and by-products generated to treat wastewater. From an economic and social development perspective, it is crucial to find milder reaction conditions and effective catalysts to remove various pollutants from wastewater. Since 1972, heterogeneous photocatalysis has been rapidly studied and applied in various areas such as water splitting, water/air purification, CO_2_ reduction, and N_2_ fixation. Photocatalysis with mild conditions, a simple process and green technology, can degrade organic pollutants contained in wastewater into water, carbon dioxide or other small molecules, and reduce or oxidize inorganic pollutants to harmless substances [9,10]. However, the catalyst is prone to self-etching in photocatalysis due to its own instability of deactivated photocatalysts. It has been widely investigated for the construction of heterojunction structures, doping, defect fabrication to improve photocatalytic performance, and the restoration of efficient photocatalytic performance by oxidative reduction of deactivated photocatalysts for recycling.

In this work, we overview the recent advances in photocatalytic removal of several common categories of water pollutants and emphasize the design and development of those materials. By reviewing the current research progress, we hope to provide directions for the modulation modification of photocatalysis, intermediates, photocatalytic mechanism and design of photocatalytic reactors, and to provide forward-looking ideas and prospects for the future development of complex structured photocatalysts and their composite systems in water waste treatment.

## 2. Basic Principles

**Figure 1:**
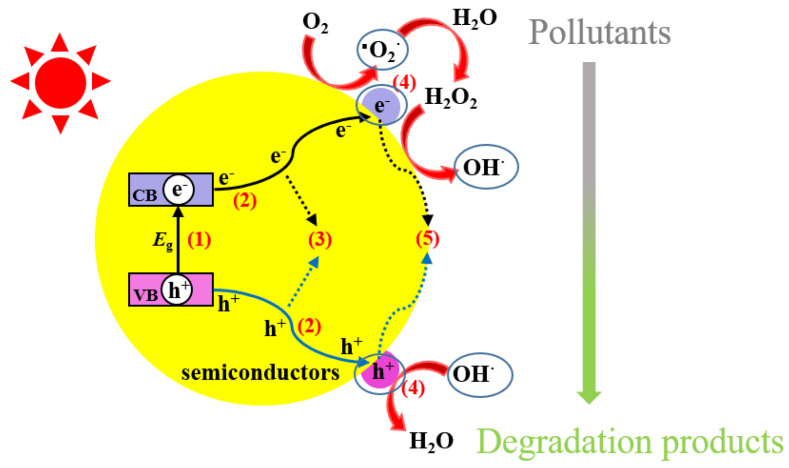
Photocatalytic processes over a heterogeneous photocatalyst.

As shown in Figure 1, the first step for a photocatalytic process is the excitation of photogenerated electron-hole pairs with sufficient energy (equal to or higher than the band-gap energy (*E*_g_) of the semiconductor). In other words, the excitation of electrons (*e*^−^) in the valence band of the semiconductor subsequently transfers to the conduction band, leaving holes (*h*^+^) behind in the valence band. Therefore, a photocatalyst with a narrower band gap is in favor of capturing more visible-light photons. The second step shows the separation of photogenerated electrons and holes. However, the bulk charge carriers undergo a recombination step with the production of phonons or heat, resulting in the reduction of the number of excited charge carriers. Electrons and holes can participate in various surface chemical reactions, while these charge carriers may also be combined on the surface. Among them, the photogenerated electrons are widely considered as a reductant for directly reducing some heavy metals ions. The separated holes may react with a hydroxyl ion (OH^−^) or a water molecule to produce hydroxyl radicals (^·^OH), and also directly participate in the oxidative decomposition due to their strong oxidizability, which is the primary pathway of production of ^·^OH. In addition, the separated electrons can react with dissolved oxygen in water to produce superoxide radicals (^·^O_2_^−^); upon further reaction, the decomposition produces ^·^OH. These contaminants in water are firstly adsorbed on the surface of the catalytic material, which increases the charge mobility and further enhances its redox ability, and then a series of chemical reactions occur with the active species generated by the catalyst to obtain the degradation products. The redox reactions mentioned above are listed below (Equations (1)–(9)): A similar photocatalytic process can also occur in the so-called photo-Fenton reaction. This process is the generation of additional ^·^OH radicals from Fenton reagents (H_2_O_2_ and Fe^2+^) under UV-Vis radiation (λ < 600 nm) through two additional reactions: (i) the photoreduction of Fe^3+^ to Fe^2+^ ions as shown in Equation (10) and (ii) the photolysis of peroxides by shorter wavelengths Equation (11) [11].

(1) $$\mathrm{Semiconductor}+\mathrm{hv}\to \;h^{+}+e^{-}$$

(2) $$e^{-}+O_{2}\to {\;}^{\cdot }O_{2}{}^{-}$$

(3) $${}^{\cdot }O_{2}{}^{-}+H^{+}\to {\;}^{\cdot }OOH$$

(4) $$2^{\cdot }OOH\to \;O_{2}+H_{2}O_{2}$$

(5) $$H_{2}O_{2}+{\;}^{\cdot }O_{2}{}^{-}\to {\;}^{\cdot }OH+OH^{-}+O_{2}$$

(6) $$H_{2}O_{2}+hv\to \;2^{\cdot }OH$$

(7) $$h^{+}+H_{2}O\;\to {\;}^{\cdot }OH+H^{+}$$

(8) $$h^{+}+OH^{-}\to {\;}^{\cdot }OH$$

(9) $$\mathrm{Pollutants}+{(}^{\cdot }OH,\;h^{+},\;e^{-},{\;}^{\cdot }OOH\;or{\;}^{\cdot }O_{2}{}^{-})\;\to \;\mathrm{degradation}\;\mathrm{products}$$

(10) $$F{e(\mathrm{OH})}^{2+}+hv\to Fe^{2+}{+}^{\cdot }OH;\;\lambda <580\;nm$$

(11) $$H_{2}O_{2}+hv\to 2\cdot OH;\;\lambda <310\;nm$$

For single photocatalysts, the high electron/hole recombination rate significantly inhibits the photocatalytic performance. The study of improving photocatalytic activity by inhibiting their recombination has received increasing attention from researchers. Various modification strategies have been developed for addressing the drawbacks above. Metal doping and morphology control can prevent complexation to some extent, and the integration of plasma excitonic elements and upconversion effects into materials for photocatalysis greatly expands the absorption and utilization of light, which provides important implications for the development of new efficient photocatalysts with broad-spectrum absorption properties. In addition to these approaches, some other approaches have been studied and reported in biphasic semiconductors, such as the formation of heterojunctions between semiconductors and the construction of external circuits using photocatalytic effects, both of which can effectively improve photocatalytic performance [12,13,14].

## 3. Removal of Organic Compounds

There are a considerable number of different types of organic pollutants in water. These pollutants can be categorized into dyes, phenolic compounds, surfactants, organohalides, hydrocarbons, plasticizers etc. [15] These organic pollutants are chemically stable, toxic and even carcinogenic, and refractory to decompose in water [16,17]. Frank and Bard [18] early committed to the photodecomposition of cyanide in water on TiO_2_. Then Carey reported catalytic degradation of polychlorinated biphenyls by TiO_2_ under UV light, leading to a foundation for the research of photocatalysis [19]. Soon afterwards, Ollis et al. [20,21] found that halogenated organic compounds such as trichloroethylene and trichloromethane would induce photocatalytic oxidative decomposition in a TiO_2_-sensitized system and formally proposed the oxidative decomposition function of semiconductor photocatalytic materials for organic pollutants, which became one of the most active fields of research in the last half-century. In general, an armory of semiconductors such as TiO_2_ [22], ZnO [23], Fe_2_O_3_ [24], C_3_N_4_ [25], and bismuth-based semiconductors [26] have been good candidates for degrading a wide range of organic pollutants into readily biodegradable compounds or less toxic molecules, which are eventually further mineralized into harmless CO_2_ and H_2_O.

### 3.1. Dyes

More than 100,000 commercially available dyes are essential in industrial processes for a wide range of products [27]. Most dyes are water-soluble, not readily biodegradable, and potentially harmful to the ecosystem, such as Rhodamine B (RhB), Methyl Orange (MO) and Methylene Blue (MB).

Duan and co-workers [28] synthesized Ag@AgCl@MIL-100(Fe)/CCF, consisting of carboxymethylation of cotton fabric as a scaffold, and in situ synthesis of MOF. During the photocatalysis process, photogenerated *h*^+^ and H_2_O forms potent oxidizing hydroxyl radicals, and superoxide ions are formed via the reactions of photogenerated electrons and O_2_. Therefore, these radicals, with a strong oxidizing power, become the culprits of MB dye degradation. It is observed that photocatalysts achieved excellent recyclability and outstanding simultaneous removal efficiency of the soluble dyes. Nguyen et al. [29] reported TiO_2_/ZnO/rGO composites’ impact on the degradation of MB, RhB, MO and proposed the degradation mechanisms and pathways under UV irradiation. Furthermore, the heterojunction of TiO_2_/ZnO/rGO materials can exhibit excellent photocatalytic practical applicability, stability, and recyclability compared to the single materials. This is attributed to the improved separation efficiency of the charge carriers, large surface area, narrow bandgap, and high adsorption capacity of the dye. Pan et al. [30] indicated that the BiFeWO_6_/α-AgVO_3_ composite with 1 wt.% of BiFeWO_6_ could significantly contribute to higher RhB removal than that of pure α-AgVO_3_ and BiFeWO_6_. With respect to pure MoS_2_ or SnO_2_, the SnO_2_-MoS_2_ nanostructures exhibited a noticeable enhancement for the photocatalytic degradation of MB and RhB, which contributed to the high specific surface area and enhanced absorption of visible light [31]. Smith et al. focused on the morphology of immobilization of ZnO on PALFs and the photocatalytic performance of ZnO/PALFs, which depends on their morphology in the removal of Congo red containing wastewater. The catalyst showed a high performance (>95%) and reusability for Congo red degradation under UV/vis irradiation conditions [32].

### 3.2. Petroleum Hydrocarbons

Petroleum hydrocarbon pollutants are persistent priority pollutants containing alkanes, olefins, and polycyclic aromatic hydrocarbons [33]. As is well known, the marine environment is considered the ultimate and largest sink of petroleum hydrocarbon pollutants. Accordingly, it has become a critical issue to effectively treat petroleum hydrocarbons in water [34].

**Figure 2:**
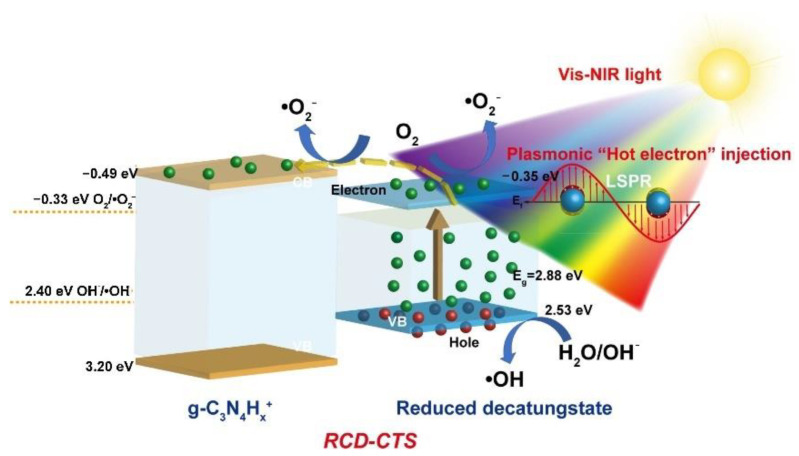
Mechanism of RCD-CTS photocatalyst under Vis-NIR light excitation [37]. Copyright, 2020 Elsevier Inc.

The biochar-supported K-doped g-C_3_N_4_ composites exhibited excellent photocatalytic naphthalene degradation activity (82.19%) under visible light irradiation due to their large number of surface hydrophilic functional groups, enhanced visible light absorption, and inhibited the recombination of photogenerated. It is observed that the photocatalytic degradation rate basically remained unchanged after five cycles [35]. Younesi et al. [36] found that nano-TiO_2_/Fe-ZSM-5 presented efficient photocatalytic removal of organic pollutants from petroleum refinery wastewater. The maximum chemical oxygen demand(COD) removal efficiency of 80% after 4 h of UV irradiation at a photocatalyst concentration of photocatalyst concentration of 2.1 g L^−1^, pH of 4, temperature of 45 °C. Two-dimensional ultrathin g-C_3_N_4_ nanosheets have high specific surface areas, short carrier migration distances and controllable electronic structures, which have good photocatalytic oxidation removal ability for petroleum hydrocarbons in aqueous solution. Yang et al. [37] proposed a distinctive visible-NIR-light-responsive decatungstate charge-transfer salt hybrid material through the assembly of g-C_3_N_4_H*_x_*^+^ cation and decatungstate anion, which displayed the efficient separation of charge-carriers by the local surface plasmon resonance. Consequently, it possessed an excellent photocatalytic activity and a good reusability for the removal of petroleum hydrocarbon (Figure 2).

### 3.3. Phenolic Compounds

Phenols in wastewater arise from a large number of industrial processes such as refineries, manufacturing of paints, pharmaceuticals, and petroleum production, are highly soluble in water, acutely toxic and biologically recalcitrant [38]. In the photocatalytic process, the main reaction site where phenol and its chlorophenol and nitrophenol derivatives are broken is the bulk liquid, and the attack of hydroxyl radicals on the cyclic carbon leads to various oxidation intermediates. Hydroquinone, catechol and p-benzoquinone were reported to be the main intermediates formed during the photocatalytic degradation of phenol. The intermediates of the reaction such as chlorohydroquinone, 4-chlorocatechol, and resorcinol are eventually converted to acetylene, maleic acid, carbon monoxide, and carbon dioxide. Chlorophenols are moderately toxic water pollutants and are suspected to be carcinogenic. The main by-products detected during its photocatalytic degradation are 4-nitrocatechol, benzoquinone, hydroquinone, and some organic acids [39].

**Figure 3:**
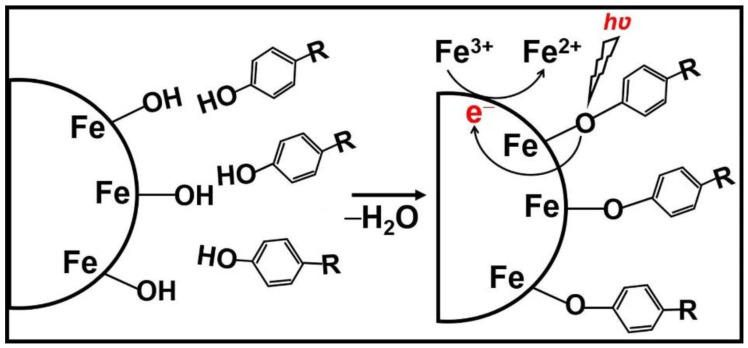
Schematic illustration of the ligand-to-metal charge transfer mechanism by chlorinated phenolic compounds in the Fe_3_O_4_ NPs system [43]. Copyright, 2020 Elsevier Inc.

Darabdhara et al. [40] first used nanomaterials to detect degradation environmental pollutants. This study reported the successful design of Au and Ni core-shell nanoparticles of size < 8 nm on rGO through the solvothermal route, and the Au@Ni/rGO nanocomposites showed excellent photocatalytic degradation for the degradation of phenol, 2-chlorophenol and 2-nitrophenol under natural sunlight irradiation with degradation rates exceeding 87%. In order to measure the catalytic activity, this nanocomposite retains cyclic stability after six cycles of use due to its unique magnetic properties. Cu doped nickel oxide nanocatalysts exhibited a phenol removal rate of about 85.7% from dermal industrial wastewater within 150 min [41]. This is because, on the one hand, doping of Cu can induce oxygen vacancy, which can be used as the NiO surface site for water dissociation. It can also effectively improve the efficiency of electron-hole separation. On the other hand, electrons captured at the Cu^2+^ site can generate superoxide radical anions (^·^O_2_^−^) through the oxidation process with the adsorbed O_2_, and the holes can react with the contained H_2_O to produce hydroxyl radical (^·^OH). The synergistic effect of hydroxyl radical and superoxide radical leads to the degradation of phenol. Besides, p-n heterostructures of CuO-TiO_2_ with tunable compositions have been synthesized via simple combining and have exhibited good photocatalytic degradation performances over both methylene blue and 4-Nitrophenol under visible light irradiation, which resulted from their particle-fiber architecture, staggering band structure, and efficient charge separation [42]. Defect engineered Fe_3_O_4_ nanoparticles with magnetic properties have exhibited high photocatalytic activity for phenolic compounds, mainly the adsorption sites with phenolic compounds provided by defect engineering on Fe_3_O_4_ (Figure 3) [43]. Yin et al. demonstrated that the cobalt-based ZIF complex coordinated with the defective TiO_2_ exhibited the highest activity for photocatalytic degradation efficiency of biphenyls 4, which was due to the more appropriate redox potential and the improved charge carriers’ separation [44]. In addition, Ph-F has been well used as one of the most suitable pre-treatment/processing systems. For example, Alessandra et al. [45] prepared magnetic particles coated with different amounts of humic acid. The iron morphology on the surface plays a crucial role in the activation process of hydrogen peroxide, which promotes Fenton and photo-Fenton-like processes investigated by using 4-chlorophenol as a standard substrate.

**Figure 4:**
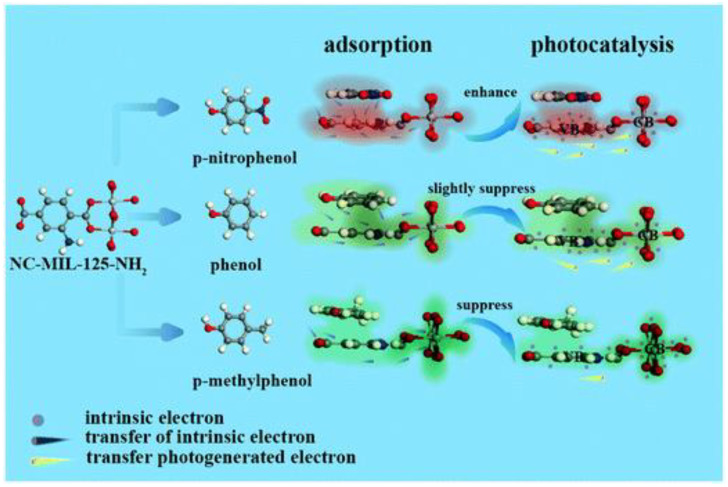
Schematic of the electron-density-induced structure-performance relationship between phenolic molecules and NC-MIL-125-NH_2_ in adsorption-photocatalysis [47]. Copyright, 2020 Royal Society of Chemistry.

A large number of studies have currently focused on improving photocatalytic efficiency through the preparation and modification of catalytic materials. Still, the molecular structure of phenolic compounds also affects the photocatalytic activity. By investigating the effect of the position and the number of phenolic substituents on the photocatalytic degradation reaction activity, it was found that the weak drawing group (-Cl) benefited the most, while substituents with a high withdrawing or donating ability decelerated the reaction, and the significant blocking effect of 1,4-Benzoquinone also confirmed that ^·^O_2_^−^ radicals were the main active species in the photolytic decomposition of phenolic compounds. Xie et al. [46] put forward ^·^O_2_^−^-mediated nucleophilic and electrophilic reaction pathways in photocatalytic reactions for the first time. It should be noted that during the degradation of p-chlorophenol, the electrophilic and nucleophilic properties of ^·^O_2_^−^ occur simultaneously, resulting in the highest degradation rate. It was proposed that the photocatalytic activity of nanocage-like MIL-125-NH_2_ was enhanced by the adsorption of electron-absorbing pollutants and were inhibited by the adsorption of electron-donating pollutants, which provided a reasonable research basis for the photocatalytic degradation of phenolics by MOF materials (Figure 4) [47].

## 4. Removal of Heavy Metals

Heavy metal ions are important for metabolism, but exhibit toxicity with high concentrations. With the development of metallurgy, mining, nuclear energy and chemical manufacturing, large amounts of toxic heavy metal ions are produced, exhibiting a severe threat to the surface and underground water resources. In living organisms, heavy metal ions are particularly capable of binding to nucleic acids, proteins and small metabolites, destroying organic cells in the body and causing fatal health problems. Since heavy metal ions cannot be biodegraded, they will be enriched in humans and animals through the food chain and drinking water [48]. Therefore, it is necessary to eliminate such hazardous heavy metals, commonly including Cr, Hg, Cd, Ni, Zn, and Mn ions, in wastewater before discharging them into the ecosystem. Photocatalytic removal of heavy metal ions in water can be achieved by reducing toxic high-valence heavy metal ions into low-valence ions or zero-valence metals.

### 4.1. Chromium (Cr)

Chromium ions and their compounds are released into the environment as carcinogens through oxygen anions (CrO_4_^2^^−^, Cr_2_O_7_^2^^−^ or HCrO_4_^−^) and cations (Cr^3+^), where they are released into the environment as carcinogens and directly harm human skin and internal organs [49,50]. The net reaction in acid aqueous solutions for Cr (VI) reduction is (Equation (12)):

(12) $$2\mathrm{Cr}O_{7}{}^{2-}+16H^{+}\to 4Cr^{3+}+8H_{2}O+3O_{2}$$

and at neutral aqueous solutions (Equation (13)):

(13) $$2CrO_{7}{}^{2-}+16H^{+}\to 4Cr^{3+}+8H_{2}O+3O_{2}$$

**Figure 5:**
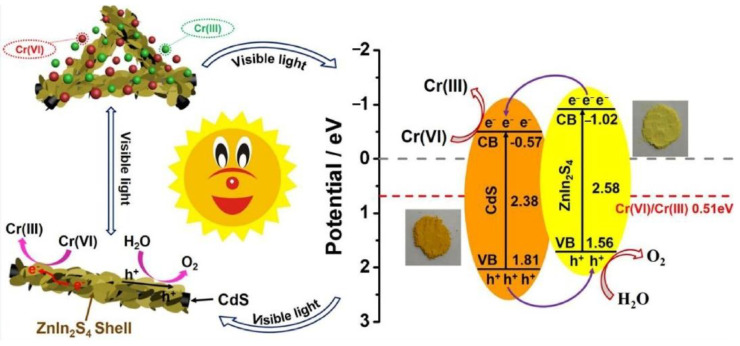
Schematic illustration of the reaction mechanism for reduction of Cr(VI) over 3D ZnIn_2_S_4_/CdS composite under visible light irradiation [54]. Copyright, 2018 Elsevier Inc.

Photocatalysis has been applied in the treatment of chromium in water. TiO_2_-ZrO_2_ has been used for the removal of heavy metal ions, exhibiting relatively highly chemical stability and excellent sorption characteristics. As reported, this material can efficiently degrade Cu(II) and Cr(VI) in one step and exhibited the high removal rates of Cr(VI) (100%) and Cu(II) (91%) after four cycles [51]. Organic-inorganic hybrid PW12/CN@Bi_2_WO_6_ composite exhibited enhanced absorbance of photons and promoted charge transfer, and achieved a removal rate of 98.7% for Cr(VI) [52]. Zhang et al. used nanocomposites consisting of freeze-dried carbon quantum dots and CdS nanosheet precursors, resulting in 94% efficiency for photocatalytic reduction of hexavalent Cr (VI) [53]. ZnIn_2_S_4_/CdS heterostructure also exhibited increased visible photocatalytic activity and durability for Cr(VI) reduction (Figure 5) [54]. The new Mn_3_O_4_@ZnO/Mn_3_O_4_ heterojunction was used for Chrome VI reduction, the process of studying the removal mechanism showed that Cr (VI) was reduced to Cr (III) by photocatalysis, and that Cr (III) was further removed by adsorption and the results showed the Cr (VI) reduction efficiency of 94.0% within 70 min under simulated sunlight irradiation [55].

### 4.2. Lead (Pb)

Lead (II) pollution is mainly anthropogenic and comes from municipal sewage, mines, and chemical production. Lead is associated with several toxicological effects on human health and behavior change, and even lead poisoning can be fatal. The possible photocatalysis reactions are as follows (Equations (14) and (15)):

(14) $$2Pb^{2+}+2H_{2}O\to 2Pb^{0}+4H^{+}+O_{2}$$

(15) $$2Pb^{2+}+2H_{2}O\to 2Pb^{0}+4H^{+}+O_{2}$$

**Figure 6:**
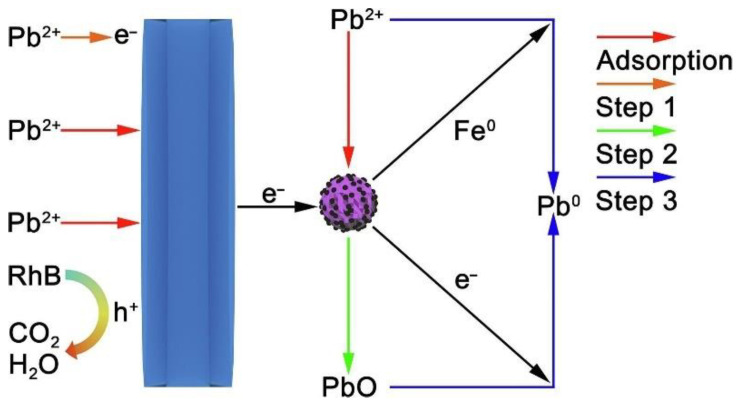
Schematic illustration of the simultaneous elimination mechanism in 1FeCTi [59]. Copyright, 2019 Elsevier Inc.

Heterogeneous photocatalysis can be a facile method to remove Pb(II) from aqueous solutions [56]. Alexander’s group [57] has successfully synthesized the superparamagnetic NiFe_2_O_4_-Pd nanohybrid. The removal efficiency of NiFe_2_O_4_-Pd against Pb^2+^ and Cd^2+^ ions were 98% and 97%, respectively, which showed higher photocatalytic activity than bare NiFe_2_O_4_. Kanakaraju et al. [58] showed that catalyst multifunctional TiO_2_/Alg/FeNPs magnetic beads had a large removal rate for a variety of heavy metal ions, the removal of mixed heavy metals, specifically Cr(III), Cu(II), and Pb(II) ions, were nearly completed at removal (>98.4%) for all three ions within 72 min. Hao et al. [59] determined that magnetic Fe_3_O_4_@C@TiO_2_ heterostructure showed a potential for capturing and removing Pb(II) (92% within 3 h). A four-step mechanism (Figure 6) was proposed that Fe^2+^/Fe^0^ can be produced by the iron oxides and *e*^−^, then Fe^0^ and *e*^−^ can reduce the adsorbed Pb (II) and immobilized PbO. Subsequently, they systematically studied the photocatalytic removal of Pb (II) on the titanate photocatalysts with different amounts of intercalated Pb (II). Enhanced photocatalytic Pb(II) removal performance of 0.10 mM Pb-Titanate was attributed to a large specific surface area, a higher adsorption affinity, and a superior number of photogenerated carriers [60].

### 4.3. Mercury (Hg)

Mercury (II) is a frequent constituent of industrial wastewater, mainly from industrial discharges such as chlor-alkali, plastics, batteries, electronics, and used medical devices. The major damage to human health is in the inhalation of mercury vapor or organic mercury ingestion through aquatic organisms, known as Minamata disease [61]. The global reaction for metallic mercury deposition is shown in Equation (16).

(16) $$Hg\left(II\right)+H_{2}O\to 2H^{+}+1/2O_{2}+Hg\left(0\right)$$

An interesting application of Hg(II) photocatalysis is the use of mesoporous α-Fe_2_O_3_/g-C_3_N_4_ nanocomposites, which showed a 4.6 times and 6.8 times higher photocatalysis activity than pure α-Fe_2_O_3_ NPs and g-C_3_N_4_ nanosheets [62]. Au-decorated TiO_2_ nanotubes exhibited the photocatalytic abatement of Hg(II) in aqueous solutions [63]. Another remarkable example is that mesoporous CuO/g-C_3_N_4_ heterostructures showed an impressive Hg (II) photoreduction rate of 628.74 µmol g^−1^ h^−1^ [64]. Kadi et al. prepared a mesoporous CoFe_2_O_4_/g-C_3_N_4_ with a large surface area (151 m^2^ g^−1^) and a tight bandgap (2.05 eV) up to the excellent Hg(II) photoreduction under visible light illumination [65].

### 4.4. Other Heavy Metals

In addition to the common heavy metal ions mentioned above, heavy metal ions such as Arsenic (As), Uranium (U), and Cadmium (Cd) are difficult to biodegrade, quickly accumulate in organisms and the environment, and are highly toxic, although their concentrations are low. Therefore, effective removal of these toxic heavy metal ions is essential for human and environmental protection [49]. For example, Ebrahimi et al. [66]. Synthesized a BiVO_4_/TiO_2_/LED system via a hydrothermal method. They observed that more than 99.97% of arsenic at pH 4.5 had been removed within 120 min using optimum conditions. Recently, nano-Fe_3_O_4_ encapsulated in a carbon sphere as the photocatalytic nanocomposite showed a higher capacity for oxidizing As(III) at pH of 3.0, and the removal efficiency approximately 70% of As(III) can be achieved at the concentration of 400 μM within 120 min [67]. Xu et al. [68] performed an Ag-doped SnS_2_@InVO_4_ hybrid system for removal of arsenic under visible light. They reported that the best results had been obtained by the optimal content of InVO_4_ (2 wt.%) at pH 6 with 97.6% removal within 120 min. Chowdhury et al. [69] also reported that Eosin Y-sensitized TiO_2_ photocatalyst showed 100% Cd (II) removal in 3 h at pH of 7.0. In addition, on the basis of ensuring good catalyst performance, the influence of environmental factors on the performance should be further investigated. For example, the solution pH not only affects the surface charge of the material but also changes the distribution of heavy metal ions, which leads to the electrostatic interaction between the catalytic material and the pollutant [70]. Therefore, the photocatalysis performance of the pollutant on the semiconductor under different environmental conditions and the potential reaction mechanism should be fully investigated to reference further research on the photocatalytic treatment of heavy metals.

## 5. Removal of Pharmaceutical

In past few years, the concern towards emerging contaminants such as pharmaceutical compounds (PCs) has increased due to their adverse impacts on the ecosystem [71]. Whereas conventional treatment methods such as flocculation, air stripping, reverse osmosis, etc., are limited in treating such compounds. Among all these processes, heterogenous photocatalysis is found to be one of the most efficient methods to degrade problematic pollutants such as antibiotics [72].

### 5.1. Antibiotics

Among PCs, more attention has been given to antibiotics as these affect the aquatic ecosystem and pose a threat to human health [73]. Antibiotics and their by-products have high toxicity, good stability, and great potential to interfere with the environment and ecological environment. A large number of antibiotics are discharged into the water environment through sewage and animal feces, causing severe water environmental problems. Therefore, it is of great significance to study the effective removal methods of these compounds in wastewater.

Isari et al. [74] successfully synthesized N-Cu co-doped TiO_2_@CNTs and combined it with visible light and ultrasonic radiography as a heterogeneous catalyst for the efficient treatment of sewage. Under optimized conditions, the removal efficiencies of 100%, 93% and 89% were obtained for sulfamethoxazole, the (COD), and (TOC), respectively. Moradi et al. [75] used the MgO/ZnO/Graphene (MZG) ternary nanocomposite to study the refractory sulfamethoxazole antibiotics in simulated wastewater compared to binary and single processes. After 120 min of sonophotocatalytic treatment, complete degradation of sulfamethoxazole antibiotic (55 mg/L) can be attained at MZG: 0.8 g/L, pH: 9.0, LED power: 90 W and US power: 250 W. The degradation efficiency of the nanocomposite decreased by up to 9.8% after six consecutive cycles of reuse.

Among the pharmaceutical compounds, tetracycline (TC) is also one of the most important antibiotics. Tetracycline forms antibiotic-resistant genes and ecotoxicity in aquatic systems, and widely exists in soil, groundwater, surface water, and even drinking water [76]. It is considered a potential hazard to human health and aquatic ecosystems. Morteza et al. [77] prepared the bare TiO_2_ and several CuO_(x)_-TiO_2_/MCM-41 nanocomposites with different CuO contents by the hydrothermal/impregnation method. They used these catalysts to degrade TC under ultraviolet light. It was pointed out that *h*^+^ can directly oxidize the TC but ^·^O_2_^−^ and ^·^OH were effectively oxidized TC. Isari et al. [78] prepared WO_3_/CNT nanocomposites for sono-photocatalytic removal of TC by the sono-photocatalysis process. It was proposed that ultrasonic waves promoted the splitting of dissolved oxygen and water molecules into free radicals, such as ^·^OH and ^·^O_2_^−^, then oxidation radicals react with TC molecules to produce intermediate products. Levofloxacin is a common fluoroquinolone antibiotic and a potential wastewater pollutant produced by the pharmaceutical industry. Adhikari et al. [79] synthesized the MoS_2_/Ag_2_Mo_2_O_7_ photocatalyst to oxidate the pharmaceutical compound levofloxacin under visible light. An enhanced catalytic activity (efficiency of 97%) and a high stability was observed with 30 wt.% MoS_2_/Ag_2_Mo_2_O_7_. This is because the heterojunction structure can greatly improve the electron-hole separation, enhance light absorption, and increase the interfacial charge transfer efficiency to the adsorbed substrate. Wang et al. [80] synthesized the magnetic NiFe_2_O_4_/CS composite by the hot water method to activate persulfate for the elimination of levofloxacin. When the levofloxacin degradation was carried out under the optimized condition, with 0.6 g/L NiFe_2_O_4_/CS composite and 1.8 g/L persulfate being added and the initial pH being adjusting to 5, 67%, levofloxacin was degraded within 1 h. Yang’s research group found that the recycling challenge was solved by preparing different graphene oxide loaded Ag_3_PO_4_/GO film catalysts, and the degradation rate of norfloxacin was about 83.68% in 100 min and the reaction rate constant *k* was 1.9 times that of pristine Ag_3_PO_4_ [81].

### 5.2. Anti-Inflammatories

In the widespread use of anti-inflammatory drugs, these compounds have a high polarity and a strong hydrophilicity, but the absorption coefficient in the soil is low, so they are easy to survive in underground, surface, and even drinking water resources, causing great pollution to water resources. Commonly used anti-inflammatory drugs, such as ibuprofen, naproxen, diclofenac, ketoprofen, etc., are often used in drinking water treatment plants in the raw water source. It is metabolized by the human body into the aquatic environment and significantly impacts different marine species, including freshwater algae, daphnia, and fish [82]. Achilleos et al. [83] found that semiconductor photocatalysis based on titanium dioxide is an effective method for the destruction and mineralization of diclofenac in aqueous solution. Ibuprofen is also one of the most popular non-steroidal anti-inflammatory drugs, which can be used to relieve rheumatism and chronic pain [84]. Khalaf et al. [85] studied the effect of titanium dioxide and photocatalysis on removing ibuprofen in the aquatic environment. They confirmed that the TiO_2_ active thin layer immobilized on the glass substrate could be a promising tool in the protection of the environment from emerging contaminants such as ibuprofen and its derivatives. Zhang et al. [86] used a magnetic Fe_3_O_4_@MIL-53(Fe) nanocomposite for the photocatalytic removal of antibiotics, achieving a 99% degradation rate in the presence of H_2_O_2_ at the visible light irradiation time of 60 min.

### 5.3. Lipid Regulators

Among lipid regulators, metformin is the most commonly used hypoglycemic drug in treating non-insulin-dependent diabetes or type 2 diabetes. After taking, metformin will not be metabolized by the human body and will be completely discharged from the body. These compounds enter aquatic resources through various sources, causing pollution to marine resources. Carbuloni et al. [87] used TiO_2_ and a synthetic TiO_2_-ZrO_2_ catalyst to remove metformin in sewage and confirmed that photocatalysis can effectively remove metformin in water. Chinnaiyan et al. [88] studied titanium dioxide as a photocatalyst to degrade amoxicillin and metformin. The experimental study found that when the pH was 7.6, the amount of TiO_2_ was 563 mg/L, the initial pollutant concentration was 10 mg/L, and the reaction time was 150 min, amoxicillin (90%) and metformin (98%) had the highest removal rates.

## 6. Removal of Pesticides

Pesticides are used as growth regulators, defoliants, desiccants, fruit thinning agents, ripening regulators, and to prevent deterioration during storage or transportation. However, pesticides are also one of the primary sources of water pollution. All pesticides are carcinogenic and show dangerous effects [89]. Pesticides have toxicity and biological resistance. Even trace pesticides can persist and have a massive impact on the ecosystem and human health. Semiconductor photocatalysis technology has also been applied to the field of pesticide degradation, which has chemical stability and anti-biodegradation [90,91]. The semiconductor materials for photocatalytic degradation of pesticides are mainly concentrated in various metal oxides (such as TiO_2_ and ZnO) [92]. Photocatalytic degradation of dimethenamid-P herbicide was performed by mesoporous Ag/Ag_2_O-TiO_2_ p-n heterojunction under visible light. The photoactivity results showed the complete removal of imazapyr destruction after 180 min [93]. Photocatalytic degradation of profenofos and triazophos residues in the Chinese cabbage using Ce-doped TiO_2_ was also studied [94]. Results showed that degradation efficiency of profenofos and triazophos reached 53.3% and 32.1% after 1 day, respectively. The photocatalytic activities of bare TiO_2_ and Au-modified TiO_2_ for degradation of phenoxyacetic acid under UV and visible light were studied [95]. Shawky studied photocatalytic degradation of atrazine herbicide using Ag/LaTiO_3_ nanowire [96]. The results revealed that the complete photodegradation of herbicide with photocatalyst was obtained by using 2.5 wt.% of Ag loading after 40 min under visible light.

## 7. Inactivation of Microorganisms

The common wastewater microorganisms include enteroviruses that cause a variety of gastrointestinal diseases, adenoviruses that cause respiratory diseases, coronaviruses that cause diarrhoea, tracheitis, and pneumonia, and Salmonella that cause colitis, dysentery, and meningitis. Since 1985, Matsunaga et al. reported that the use of TiO_2_ photocatalysts could kill bacteria in water, and provided a new path for the inactivation of microorganisms by photocatalysis. In addition to the degradation of various organic compounds and inorganic substances, an important aspect is the ability of Reactive oxygen species (ROS) to inactivate microorganisms, and it has been proved to be an incredibly effective method for the overall treatment of water. The effect of reactive ROS on microorganisms mainly includes the following aspects: ROS destroy the coenzyme A on the cell membrane, leading to the inhibition of respiration that depends on the intact cell membrane, the reduction or loss of cellular respiratory activity, or ROS enter the cell further to oxidize nucleic acids, proteins and other macromolecules and eventually cause cell death [97]. In addition, some studies also showed that ROS oxidized the cell membrane so that the cellular outer layer destruction triggered the leakage of nucleic acids, proteins and some cations, eventually leading to bacterial cell death [98].

**Figure 7:**
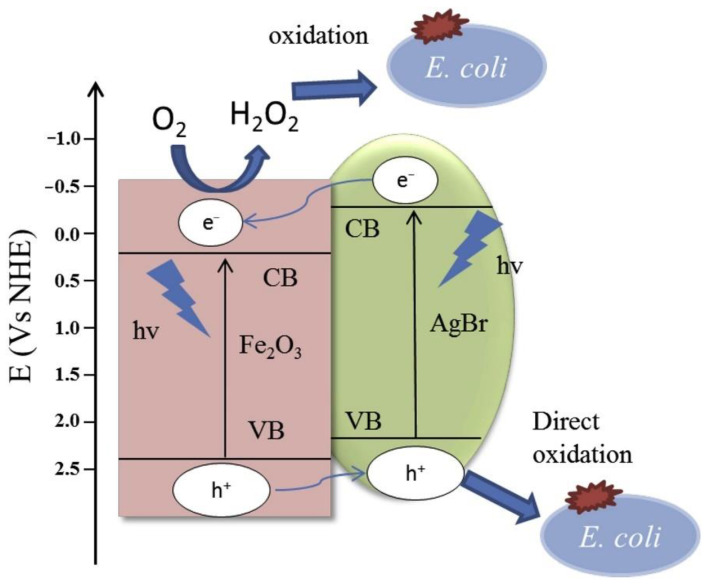
Schematic illustration of proposed mechanism of bacterial inactivation by Fe_2_O_3_-AgBr [101]. Copyright, 2016 Elsevier Inc.

Kim et al. used a Co-doped BiVO_4_ scheme to pretreat wastewater. This method can make the inactivation of *Escherichia coli* (81.3%, 5 h) and *Chlamydomonas pulsatilla* (65.6%, 1 h) [99]. Si et al. [100] reported the photocatalytic inactivation of *E. coli* by g-C_3_N_4_@Co-TiO_2_ nanofibrous under visible light irradiation. The results showed that the inactivation of *E. coli* displayed 6 log of bacterial cells reduction after 90 min. Wong et al. [101] recently investigated the effects of different physicochemical factors including photocatalyst concentration, solution pH, temperature, and inorganic ions of magnetic Fe_2_O_3_-AgBr under LED lamp against inactivating both Gram-negative (*E. coli*) and Gram-positive (*Staphylococcus aureus*) bacteria. Further study of the mechanism (as presented in Figure 7) showed that the oxidation of H_2_O_2_ generated from the CB of Fe_2_O_3_ and the direct oxidation of *h*^+^ of AgBr can still contribute to the bacterial inactivation. Matsuda et al. [102] prepared an efficient photocatalytic nanocomposite (Co_x_Ni_1−x_Fe_2_O_4_; x = 0.9/SiO_2_/TiO_2_/C-dots) through a layer-by-layer method, and nanocomposite displayed an inhibition against *E. coli* of about 80.47% and its repression to *Candida* species reached 78.54%. To provide a short summary, the most representative examples of the publications analyzed above are listed separately in Table 1. The information reported includes the characteristics of wastewater being treated, the materials used, the working conditions and the catalytic activity to obtain the most relevant conclusions. This information is discussed in detail in the following sections.

## 8. Design of Photocatalytic Reactors

In recent years, although there have been advances in the field of heterogeneous photocatalysis, a rational design of photocatalytic reactors is a deciding factor in the success of industrial application. Photocatalytic reactors play an extremely important role in the photocatalytic water treatment industry [103]. Choosing a suitable reactor can speed up the rate of wastewater treatment and effectively save energy. At present, most photocatalytic reactions are limited to the experimental stage and are difficult to put into practice in industry, partly because of the catalysts themselves, and mainly because most of the catalysts do not have suitable reactors for industrial amplification. The photocatalytic reactor uses light as the reaction energy, so the utilization efficiency of light source and the mass transfer efficiency of light in the reactor should be considered. Placement of the catalyst into the reactor will increase the contact area, catalytic performance, accelerate the reaction rate, and optimize the photocatalytic reaction. Therefore, the development of a new photocatalytic reactor is also an important way to accelerate the application of photocatalysis in practice.

Photocatalytic reactors can be divided into fluidized-bed reactors and fixed-bed reactors according to the existing form of catalyst. Fluidized bed reactors are the catalysts directly loaded on the granular carrier suspended in the solution to be treated. They have a large surface area, a small mass transfer restrictions, and a fast reaction rate, but the catalyst is difficult to recover and easy to agglomerate, resulting in blockage, meaning that industrial amplification is very difficult [104]. Although the fixed bed reactor has a smaller surface area than the fluidized bed reactor, it is easy to separate the catalyst from the liquid and the catalyst can be recycled, hence it has great industrial development prospects. At present, the choice of catalyst carrier and fixing technology are the main barriers to anindustrial amplification of fixed bed catalysts [105].

Additionally, the scale-up of a photoreactor requires the development of a mathematical model that include the enclosure of different sub-models [106]. Photocatalytic reactors have been modeled both for gaseous and liquid phases using computational fluid dynamics (CFD). In the literature, different authors developed CFD models for the scale-up of photoreactors and the treatment of wastewater streams considering model pollutants, such as oxalic acid, phenol, poly(vinyl alcohol), tributyl phosphate, tri(2-chloroethtyl)phosphate, rhodamine B, and methylene Blue, etc. In CFD modeling, the light source emission is typically represented by a linear source model, which could be good for many reactor configurations, but has also some limitations.

**Figure 8:**
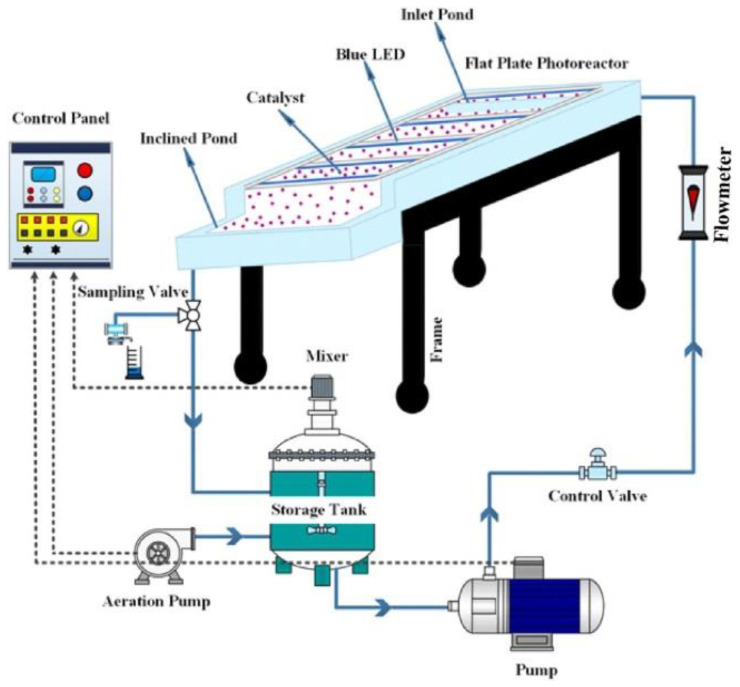
Schematic diagram of the flow-loop thin film slurry flat plate photoreactor [107]. Copyright, 2020 Elsevier Inc.

Bahmani et al. [107] synthesized BiOI/BiFeO_3_/UiO-66(Zr/Ti)-MOF complex as a novel ternary separable visible light photocatalyst. The introduction of the introduction of BiFeO_3_ perovskite and BiOI on the UiO-66(Zr/Ti) surface improves the separation and migration rate of photo-induced charges and thereby boosts the photocatalytic efficiency, in a thin-film slurry flat photoreactor (as presented in Figure 8) with a continuous flow loop illuminated by blue light-emitting diodes (LEDs). An inclined cell 22.5 cm long, 2.5 cm wide and 5 cm high with a 2 cm diameter hole was embedded at the end of the reactor to allow better drainage of the solution for effective separation. The used photoreactor was adjusted at a moderate slope (about 5) in a horizontal axis. A nanoscale roughness was created on the reactor surface, resulting in a uniform liquid film over the entire reactor surface. The reactor has sufficient stability, high photocatalytic activity, long-term durability, regeneration and reusability, and easy separation.

Meng et al. [108] designed a palladium-vanadium tetroxide glass bead-filled photoreactor, which is 132% faster than the flat plate reactor and more energy efficient in the photocatalytic degradation of phenol. The excellent performance of the reactor is mainly attributed to the highly exposed catalyst surface area, high mass transfer coefficient and the effective transfer of photons and reactants to the catalyst surface, but the cost of the reactor is much higher than TiO_2_. Qin et al. [109] prepared oriented TiO_2_ nanotubes on titanium foil by one-step hydrothermal synthesis. The adsorption and photocatalytic performance of TiO_2_/TiO_2_ foil catalyst in formaldehyde degradation was studied in detail by a self-made experimental device. TiO_2_/TiO_2_ foil catalysts can be directly designed as self-supporting ring reactors that degrade formaldehyde at least twice as efficiently as commercial TiO_2_ dioxide-based catalysts. Uniformly morphological and oriented TiO_2_ nano-wires on the Ti substrate were prepared by one-step hydrothermal synthesis, which can be recyclable without secondary pollution. Rahmani et al. [110] prepared TiO_2_/SiO_2_ thin films by a sol-gel method, which were coated on the inner wall of the outer shell of the annular photoreactor and heat-treated at 400 °C. The tubular photoreactor is used to degrade oil in aqueous solution. The results show that the photoreactive agent has excellent performance in degrading oil pollution. The optimal performance conditions of the photocatalytic reaction in the ring photoreactor were studied. The best results were obtained at a starting n-alkane concentration of 500 ppm and a pH equal to 5, under which the conversion rate of n-alkane was about 85%.

Above all, it is worthwhile to note that several parameters, such as pH, temperature, oxygen concentration, and concentration of ion scavengers need to be considered in the design of photocatalytic reactor. The development prospect of photocatalytic water treatment is great, and more reactors with an optimized model and excellent performance are worth exploring.

## 9. Conclusions and Prospects

In conclusion, this review summarized recent photocatalysis application developments in water treatment. Firstly, the mechanism of photocatalytic oxidation process was introduced, and then the recent advances in photocatalytic removal of several common categories of water pollutants were displayed in detail, combined with some novel photocatalysts. By the synergistic effect of composite components, the strategies of photocatalytic performance improvement were pointed out. Although some significant advances have been made in recent years, the degradation efficiency and reuse utilization are still low and cannot be applied in practice. From this review, it is clear that each step of the photocatalytic process, including charge excitation, separation, transport, adsorption, and surface reaction of the semiconductor, has a significant impact on the photocatalytic efficiency. In addition to the performance of the catalyst itself, the degradation concentration, pH, temperature, the charged nature of the pollutant, the reactor, and the light source lamp are also very important to achieve a maximum efficiency. Therefore, all factors should be considered and carefully optimized when designing and manufacturing multifunctional semiconductor photocatalysts for the photocatalytic treatment of organic pollutants. To realize photocatalysis of various pollutants to a more practical level, the following aspects must be considered:

(1) Better understanding of the photocatalysis of various pollutant mechanisms

The finding of the rate-determining steps in photocatalysis water treatment is favorable for the design and fabrication of highly efficient and selective photocatalysts. Thus, theoretical calculations and computational methods have made it an in-depth study to investigate surface transformations at the molecular level during photocatalytic degradation. In addition, theoretical calculations and experiments should be combined to provide deeper insights into the reaction interface.

(2) Rational design of the catalysts and the photochemical system

The catalytic activity toward photocatalytic degradation processes is extremely influenced by the physicochemical properties of materials. It has been demonstrated that catalysts with a lower dose and faster kinetics can reduce the process costs and result in an intensification of the photocatalytic degradation processes. Especially, crystal facet engineering, defect engineering, size control, heteroatom doping, semiconductor composite modification, and surface tethering are highly expected to boost the photocatalytic degradation ability. Meanwhile, the effects of various factors such as photocatalyst concentration, temperature, light source, solution pH, and inorganic ions on the efficiency of photocatalytic water treatment should be comprehensively investigated, and the optical stability of semiconductors should be fundamentally improved to prevent corrosion in the actual photocatalytic water treatment. In addition, the efficiency of light source utilization and the efficiency of light transfer in the reactor are essential for the application of photocatalytic technology in practice through the design of the reactor efficiency.

(3) Advanced characterization techniques for photocatalytic water treatment

In recent years, the exploration of the species and number of active species produced during catalyst degradation have played a crucial role in elucidating the reaction mechanism. For instance, the combination of mass spectrometry and NMR techniques to deepen the exploration of reaction intermediates is an effective way to deeply explain the transformation and removal of pollutant molecules on the surface of the material. Moreover, in the case of phenol, the degradation process is quite complex, and the intermediate products of the degradation process, such as o-diphenol, m-diphenol, quinones, and even aldehydes, are more toxic than phenols. Therefore, an in-depth study of the intermediates of the degradation process is essential for revealing the reaction mechanism, gaining an insight into the effects of various factors, controlling the reaction process effectively, and selecting the optimal conditions.

(4) Realization of photocatalytic wastewater treatment in practice

There are a couple of bottlenecks in terms of the photocatalysis powders for practical water disinfection, including particle aggregation at high concentrations and difficult separation of the photocatalyst from the treated water. Although many factors are considered in the photocatalytic water treatment experiments, they mostly involve laboratory configurations of simulated single pollutant wastewater, which still involves a large degree of complexity compared to real industrial or natural wastewater. Further development of a rational photocatalytic system is essential, including reactor design to optimize separation efficiency, photocatalyst optimization, and immobilization for recycling.

## Figures and Tables

**Table 1:** Photocatalytic removal of several common categories of water pollutants.

Treatment System	Classification	Characteristics	Materials	Pollutant	Light Source	Con. (mg/L)/(cfu/mL)	Volume (mL)	Irradiate Time (h)	Eff. (%)	Ref	Immobilized
**Organic compounds**	**Dyes** Water-soluble, not readily biodegradable, harmful to the ecosystem		Ag@AgC@MIL100(Fe)/CCF	MB	Vis light (500 W)	20	40	2/3	99.2	[28]	Yes
			TiO_2_/ZnO/ rGO	MB, RhB, MO	UV/simulated solar illumination (300 W)	20	1000	2	99.6 99.2 99.4	[29]	No
			BiFeWO_6_/ α-AgVO_3_	RhB	Vis light	0.01 mM	50	3/2	90.4	[30]	No
			SnO_2_-MoS_2_	MB, RhB	Vis light (200 W)	20	100	2	96.4 93.1	[31]	No
			ZnO/PALFs f	congo red	Vis light (300 W)	20	10	5	>95	[32]	Yes
	**Petroleum hydrocarbons**		K-doped g-C_3_N_4_	Naphthalene	Vis light (200 W)	20	100	3	82.2	[35]	No
			g-C_3_N_4_H_x_^+^	N-tetradecane	Vis light (300 W)	5000	10	4	87.3	[37]	No
	**Phenolic compounds**	Highly soluble in water, acutely toxic, biologically recalcitrant	Au@ Ni/rGO	Phenols	Sunlight	1000	-	7/2	87.7	[40]	No
			Cu-NiO	Phenols	UV–Vis (150 W)	Real effluents	-	5/2	85.7	[41]	No
			CuO-TiO_2_		UV light (96 W)	15 mM	100	1	100	[42]	No
			TiO_2_-x @ZIF-67	BPA	Vis light	50	-	1	95.3	[44]	No
**Heavy metals**	**Cr**	Carcinogens, harm human skin and internal organs	TiO_2_-ZrO_2_	Cr(VI)	UV light	0.5		1/12	100	[51]	No
			PW12/CN@ Bi_2_WO_6_	Cr(VI)	Simulated xenon light (1000 W)	20	50	3/2	98.7	[52]	No
			CdS	Cr(VI)	Vis light (300 W)	20	60	1/6	94.9	[53]	No
			ZnIn_2_S_4_/ CdS	Cr(VI)	Vis light (300 W)	50	50	1/2	100.0	[54]	No
			Mn_3_O_4_@ ZnO/Mn_3_O_4_	Cr (VI)	Sunlight (300 W)	10	200	7/6	94.0	[55]	No
	**Pb**	Toxicologica, fatal	TiO_2_/Alg/ FeNPs	Pb(II)	254 nm ultraviolet C (30 W)	20	100	6/5	99.6	[58]	No
			Fe_3_O_4_@ C@TiO_2_	Pb(II)	UV–Vis (300 W)	20	100	3	92.0	[59]	No
			TiO_2_	Pb(II)	300–450 nm (15 W)	0.5 mM	450	4	-	[56]	No
	**Hg**	High toxicity, tendency to bioaccumulate	α-Fe_2_O_3_/ g-C_3_N_4_	Hg(II)	Vis light (400 W)	100	500	1	90	[62]	No
			CuO/g-C_3_N_4_	Hg(II)	Vis light (150 W)	100	500	1	100.0	[64]	No
			CoFe_2_O_4_/g-C_3_N_4_	Hg(II)	Vis light (300 W)	100	500	1	100.0	[65]	No
**Pharmaceutical**	**Anti-biotics** Water-soluble, not readily biodegradable, harmful to the ecosystem		MgO/ZnO/ Graphene	Sulfamethoxazole	UVA (30 W)	-	200	7/2	94.4 COD	[75]	No
			TiO_2_/CuO/MCM-41	Tetracy-cline	UV light (125 W)	20	200	1	70.5	[77]	No
			MoS_2_/ Ag_2_Mo_2_O_7_	Levofloxacin	Vis light (150 W)	5	-	3/2	97.0	[79]	No
			Ag_3_PO_4_/GO film	Norfloxacin	Vis light (250 W)	15	120	5/3	83.6	[81]	Yes
	**Anti-inflammatories**	High polarity, hydrophilicity, the absorption coefficient in the soil is low	TiO_2_	Ibuprofen	Simulated solar irradiation (500 W)	10	500	4/3	87.0	[85]	No
	**Lipid regulators**	Highly soluble in water, acutely toxic, biologically recalcitrant	TiO_2_-ZrO_2_	Metformin	UV light (125 W)	1		1/2	50.0	[87]	No
			TiO_2_	Amoxicillin metformin	UV lamp (125 W)	-	200	3/2	90.0 98.0	[88]	No
**Pesticides**		Toxicity, biological resistance	Ag/Ag_2_O-TiO_2_	Imazapyr	Vis light (1 mW/cm^2^)	0.08 mM		3	100	[93]	No
			TiO_2_/Ce	Profenofos triazophos	Simulated xenon light	20	50	3/2	98.7	[94]	No
			Au/TiO_2_	Phenoxyacetic acid	Vis light	0.15	-	7	87.0	[95]	No
			Ag/LaTiO_3_	Atrazine	Vis light (300 W)	50	-	2/3	100.0	[91]	No
**Micro** **organisms**		Causing a variety of gastrointestinal diseases, adenoviruses	Co-BiVO_4_	Escherichia coli, Chlamydomonas pulsatilla	-	-	70	5 1	81.3 65.6	[99]	No
			g-C_3_N_4_@ Co-TiO_2_	Escherichia coli	Vis light (300 W)	1 × 10^6^	10	3/2	6 log inactivation	[100]	Yes
